# An Evaluation of Family-Based Treatment for OCD in Japan: A Pilot Randomized Controlled Trial

**DOI:** 10.3389/fpsyt.2019.00932

**Published:** 2020-01-09

**Authors:** Yuki Kobayashi, Ayako Kanie, Atsuo Nakagawa, Yoshitake Takebayashi, Issei Shinmei, Noriko Nakayama, Keiko Yamaguchi, Chiaki Nakayama, Naotsugu Hirabayashi, Masaru Mimura, Masaru Horikoshi

**Affiliations:** ^1^National Center for Cognitive Behavioral Therapy and Research, National Center of Neurology and Psychiatry, Tokyo, Japan; ^2^Department of Neuropsychiatry, Keio University School of Medicine, Tokyo, Japan; ^3^Clinical and Translational Research Center, Keio University Hospital, Tokyo, Japan; ^4^Department of Health Risk Communication, Fukushima Medical University School of Medicine, Fukushima, Japan; ^5^TCBT Counseling Office, Tokyo, Japan; ^6^Department of Neurology, National Center of Neurology and Psychiatry, Tokyo, Japan; ^7^National Center Hospital, National Center of Neurology and Psychiatry, Tokyo, Japan

**Keywords:** obsessive-compulsive disorder, family-based treatment, exposure response prevention, pilot randomized controlled trial, treatment

## Abstract

**Objective:** Although family involvement in the treatment of obsessive-compulsive disorder (OCD) produces a reduction in OCD symptoms and has significant effects on global functioning, few studies have focused on family intervention as part of OCD treatment in Japan. This study aims to examine the feasibility and efficacy of the family-based exposure and response prevention (FERP) program for adult patients with OCD and their family members.

**Design:** Randomized controlled pilot study.

**Methods:** A total of 18 outpatients aged 18–65 years with a primary diagnosis of OCD and one family member of each patient were randomized to an intervention group or a control group (1:1). The intervention group received the FERP program, which consisted of 16 weekly face-to-face cognitive behavioral therapy (CBT) sessions, including eight joint sessions with family members, in addition to treatment-as-usual (TAU). The control group received TAU alone. The primary outcome was the alleviation of OCD symptoms, as measured by changes in the total Yale-Brown Obsessive Compulsive Scale (Y-BOCS) score from baseline to posttreatment. Analyses were provided on an intention-to-treat basis, and linear mixed models were used to test for significant group differences.

**Results:** After 16 weeks, patients allocated to the FERP program showed improvement in OCD symptom severity, as measured by the total change score of the Y-BOCS (Hedges’ *g* = −1.58), as compared to the control group. Two patients (22.2%) in the FERP group reached remission, and five patients (55.6%) in the FERP group achieved treatment response. Clinical global improvement measured by the FAS-SR scores, K6 scores, and CGI-S scores was also observed (Hedges’ *g* = −1.35, −1.25, and −1.26, respectively) in the FERP group as compared to the control group. The dropout rate from the study was low (*n* = 2, 11.8%), and no adverse events were reported in the FERP group.

**Conclusion:** Our results suggest that FERP may be an effective program for reducing patients’ OCD symptoms.

**Clinical Trial Registration:** www.umin.ac.jp/ctr/, identifier UMIN000021763.

## Introduction

Obsessive-compulsive disorder (OCD) is a chronic disabling illness affecting 1–3% of the general population (1) and characterized by recurrent, unwanted thoughts and/or repetitive behaviors that cause notable distress and interfere in one’s daily life, including occupational or academic functioning and social activities, and also affect family relationships (2, 3).

Family accommodation is a process in which the family members of patients with OCD assist or take part in the patients’ rituals and is positively associated with more severe OCD symptoms, greater functional impairment, and reduced quality of life; in addition, it may also predict poor treatment outcomes (4, 5). More than 95% of OCD patients’ families accommodate the patients’ OCD rituals by providing reassurance, participating in compulsive behaviors, waiting for ritual completion, or avoiding OCD triggers (6). Family accommodation is often a “successful” coping measure for the patient in the short run as it provides a sense of relieving distress and facilitates quick completion of avoidance and compulsive behavior. However, “family accommodation eventually results in a vicious cycle in which patients become more likely to engage in avoidance or compulsive behaviors, and hinder them from developing more adaptive appraisals and behaviors to cope with their OCD-related distress in the long run” (3). Of note, “family accommodation for patients with clinical anxiety manifest in various forms and is often linked to specific areas of the affected family member’s anxiety” (7).

The first-line treatment for OCD includes cognitive behavioral therapy (CBT) and pharmacological treatment using serotonin reuptake inhibitors (i.e., SSRIs or clomipramine) (8). Evidence shows that family accommodation is associated with poor treatment outcomes, and this association is also observed between exposure response prevention (ERP), a type of CBT, and accommodating behaviors known to counteract treatment efficacy (9). Therefore, approaches aimed at reducing family accommodation by improving knowledge and enhancing adaptive behavioral models are important for achieving therapeutic goals (7). OCD has been reported to respond positively to treatment in the short term, but has a high relapse rate (10, 11); therefore, including the family in learning about family accommodation and capacitating them with the necessary coaching skills to guide ERP should have a positive impact on the sustainability of treatment effects in patients with OCD. It is noteworthy that clinical practice guidelines for the treatment of OCD in the UK and North America recommend family involvement (12).

Evidence shows that supplementary family interventions for adult OCD patients have better overall treatment outcomes. A meta-analysis of family involvement in psychological treatment showed that individual family-inclusive treatment of OCD had a large effect on OCD symptoms (*d* = 1.68) and global functioning (*d* = 0.98) (13). One randomized controlled trial (14) examined the efficacy of an adjunctive, brief family intervention involving two sessions to reduce family accommodation and observed a significant reduction in OCD symptoms in patients whose family members got adjunctive treatment. Another trial examined the effectiveness of a brief, family-based intervention (BFBI) as an adjunct to SSRIs versus SRI + relaxation exercise (RE) and found that the severity of OCD, family accommodation, and expressed emotion decreased significantly over time in the BFBI group as compared to those in the RE group (15). Family intervention was also found to be effective for pediatric OCD patients. The pediatric OCD treatment study for young children (POTS Jr) examined the relative efficacy of two manualized treatment programs—family-based CBT (FB-CBT) versus family-based relaxation treatment (FB-RT)—for children aged 5 to 8. The effect size of the FB-CBT on OCD symptoms was 0.84, indicating a large standardized effect size (16). In another family-based ERP study, OCD patient aged 3–8 years and one of their parents each were randomized to family-based ERP or treatment-as-usual (TAU), and a significant group effect was observed (*d* = 1.69) in the family-based ERP group compared to the TAU group (17).

Previous family interventions targeted OCD symptoms as well as family accommodation. The FB-CBT focused on providing the child and parents with “tools” to reduce OCD symptoms, such as behavioral management of the child’s OCD symptoms with differential attention, as well as modeling and scaffolding (16). Another FB-CBT focused on reducing parental accommodation immediately after treatment and parent-driven ERP at home. In these studies, both youth and parents had to attend all sessions together as a family to overcome OCD. The inclusion of parents as coaches fosters motivation, which may lead to reduced family accommodation. As such, family accommodation may mediate OCD symptom outcomes.

OCD-related family pathology is quite common in Japanese clinical settings. Over 40% of Japanese patients with OCD reported some involvement behaviors (IB) by their families to help or take part in their rituals, such as “asking family member for reassurance” (18). Such IB is a significant predictor of poor outcomes in OCD treatment (19). In the Japanese culture, an individual is expected to silently tolerate the anxiety experienced instead of verbally complaining. Therefore, compared to England, it is easier for Japanese parents to reinforce non-anxiety physical symptoms, such as cold-related symptoms, than anxiety-related symptoms (20). Additionally, family support groups and OCD information for patients’ families are very limited in Japan. Such a cultural background may result in verbal miscommunication among Japanese families and limited familial understanding of OCD and ERP. These concepts can be considered important for OCD treatment in Japan. Nonetheless, few studies have focused on family intervention for the treatment of OCD in Japan.

Given the clinical need to improve the current standard of OCD treatment in Japan through the provision of supplementary family intervention, we developed a family-based ERP program and conducted a case series study in a clinical setting (21). The program included one family member of each patient for a 16-week intervention. Results from the study indicated an improvement in the OCD symptoms and psychological distress as well as a decrease in the frequency of family accommodation post-intervention. Building upon these findings, we conducted a pilot randomized clinical trial to examine the feasibility and efficacy of the family-based ERP (FERP) program for OCD patients and their family members as an adjunct to TAU, while comparing this approach with TAU alone. We hypothesized that 1) FERP would result in greater improvement of OCD symptom severity as compared to the control group, and 2) FERP would result in improved family accommodation and family depression symptom severity compared to the control group.

## Materials and Methods

### Study Design

The present study was designed as a two-study site, assessor-blinded, randomized controlled trial with two parallel groups on a 1:1 allocation. The trial was registered in the UMIN Clinical Trials Registry (identifier: UMIN000021763) and was conducted in accordance with the CONSORT guidelines.

### Patients

Patients were recruited from the outpatient clinic at a psychiatric hospital and a university teaching hospital in Tokyo from June 2016 to March 2019. Patient screening was administered by psychiatrists in charge of the psychiatry outpatient first visit service. Patients were eligible for the study if they met the following inclusion criteria: a) diagnosis of OCD according to DSM-IV criteria; b) at least a mild severity of OCD symptoms; c) a total score ≥ 8 on the Yale-Brown Obsessive Compulsive Scale (Y-BOCS) (22); d) age between 18 and 65 years at screening; and e) able to visit the hospital more than 12 times during the study period. Exclusion criteria were: a) other primary DSM-IV Axis I disorders in addition to OCD; b) alcohol or substance use disorders within 6 months of screening; c) serious suicidal ideation at screening; d) current or past treatment with individual face-to-face CBT for OCD; e) major cognitive deficits at screening; and f) severe or unstable medical comorbidities at screening. Patients’ family members were eligible for the study if they met the following inclusion criteria: a) living/in contact with the patient at least 1 h per day; b) family member who is most involved with patient’s OCD as identified by the patient; and c) able to visit the hospital eight times during the study period. We explained the study procedures in detail and obtained written informed consent from patients and their family members, and the study was approved by the ethics committee at each study site.

### Randomization and Masking

Eligible patients and their family members were randomly assigned to either the intervention (family-based ERP + TAU = FERP group) or control (TAU only) groups with a 1:1 allocation using a central computerized registration system. This computerized system automatically randomized study participants and generated a message noting their assigned treatment. Randomization was stratified by study site with the minimization method to balance the age of the patients at study entry (<40 years, ≥40 years).

Due to the nature of the interventions, neither the participants nor the treating therapists could be masked to randomization status, but the assessors were masked as much as possible. The assessors did not participate in the treatment delivery and were prohibited from accessing any information or documents that could reveal participant allocation. Participants were instructed not to disclose their allocated treatment to the assessors during their assessment interviews.

### Interventions

#### Family-Based ERP Program

**Table 1** shows the framework of the FERP program. The goals of the program were the following: 1) ability to plan home-based ERP with the patient after participating in the program and 2) ability to assist the patient in recovery from OCD by supporting the patient as a coach or supporter without accommodating their OCD symptoms. The FERP program consisted of sixteen 60-min sessions that were conducted weekly, with an extension or abbreviation of four sessions if deemed clinically appropriate by the treating therapist (i.e., a range of 12–20 sessions in total). The program was based on the treatment manual of CBT for OCD distributed by the Japanese Ministry of Health, Labour, and Welfare (23), and each session was conducted using CBT treatment materials for OCD patients (24).

**Table 1:** Framework of the eight joint sessions of FERP with the patient and family member.

**Session**	**Goals**	**Components**	**Tools/homework**
1	• Rapport-building • Understanding of OCD and current OCD symptoms • Normalize the patient’s difficulties	• Review of OCD symptoms • Psychoeducation on CBT model and OCD	• Provide educational sheets • “Review of your OCD” • “OCD monitoring sheet”
2	• Understanding of ERP • Goal setting	• Psychoeducation on ERP and rationale for treatment	• Provide educational sheets • “Anxiety hierarchy”
3	• Understand how to support the patient during home-based ERP	• Review of examples to motivate the patient during home-based ERP	• Provide educational sheets
4	• Understanding of communication skills	• Review of communication difficulties in the patient’s family • Role-play exercise to practice communication skills	• Provide educational sheets
5	• Understanding of the FA of OCD • Identify the type of FA of the patient	• Review various types of FA • Collaboratively identify the type of FA of the patient	• Provide educational sheets
6	• Understanding how to respond to the FA	• Review the examples for responding to FA of OCD • Discuss how to respond to the patient and how to really support the patient	• Provide educational sheets • “How to respond to FA”
7	• Relapse prevention • Understanding of goal setting to overcome OCD	• Review how to set goals when OCD occurs again • Collaboratively setting long- and short-term goals after CBT	• Provide educational sheets • “Goal-setting sheet”
8	• Relapse prevention • Understanding how to negotiate a family contract to achieve the goals	• Review the examples of a family contract for OCD • Planning a family contract to achieve their goals • Review of the therapy and get feedback	• Provide educational sheets • “Family contract sheet”

A total of eight 20-min joint family sessions, which involved the patient, their family member, and the therapist, were included in the intervention. Original educational sheets developed specifically for this intervention were provided to the participants at each session. Each sheet consisted of 12–15 pages, based on the structure of the session, and included illustrations and worksheets to aid the understanding of the session.

Four clinical psychologists with doctorate or master’s degrees facilitated the FERP program. All therapists completed the 2-day general CBT and 2-day CBT for OCD workshops before the study. They had practiced CBT for OCD for a mean (SD) of 7.0 (2.0) years and had treated 22.5 (18.1) OCD patients before the study with CBT for OCD. During the intervention, they received 1-h on-site group supervision sessions every week from a skilled CBT supervisor (M.H.), with thorough reviews of the cases and detailed feedback to maintain competence and adherence to the study protocol. Two clinical psychologists independent of treatment delivery rated adherence using competence and compliance scales developed for this study; three sessions were randomly sampled for this evaluation.

For safety reasons, the therapist had to immediately notify the treating psychiatrist of any serious adverse events. In addition, at each visit, the therapist asked the patient about any worsening of symptoms over the past week.

#### Treatment as Usual

For this clinical trial, participants allocated to both the FERP and control groups continued treatment as usual with their treating psychiatrist. If they received pharmacotherapy, there were no restrictions placed on the pharmacotherapy provided, except concurrent individual face-to-face CBT for OCD and electroconvulsive therapy. Pharmacotherapy was based on the practice guidelines for OCD patients published by the American Psychiatric Association Practice Guidelines (8). Treating psychiatrists were not involved in the delivery or supervision of this study.

### Outcomes

The primary outcome was the change in OCD symptoms, which was measured by the total score of the Japanese version of the Yale-Brown Obsessive-Compulsive Scale (Y-BOCS) (25) during clinical interviews at baseline and post-intervention. The Y-BOCS were assessed by three psychologists with doctorate or master’s degrees. The following three procedures were utilized by the assessors for their training: 1) watching the Y-BOCS training video published in Japan; 2) assessing eight recorded OCD cases that we had already assessed; and 3) assessing two or three OCD cases not included in this study. The inter-rater reliability for the total Y-BOCS score was 0.94 (95% confidence interval, 0.86–0.98). The secondary outcomes included treatment response (≥35% reduction in baseline Y-BOCS score), remission (≤12 on the Y-BOCS score), participant-rated measure of depressive symptoms [Beck Depression Inventory-II (BDI-II)] (26), degree of psychological distress [Kessler Psychological Distress Scale (K6)] (27), level of functional impairment [Sheehan Disability Scale (SDS)] (28), level of health-related quality of life [EuroQol (EQ-5D-3L)] (29), the frequency of family accommodation [Family Accommodation Scale for OCD Self-Rated version (FAS-SR)] (30), and patient-rated version (FAS-PV). The patients were also asked to assess their global impression of severity [Patient Global Impression-Severity (PGI-S)] (31); in addition, the assessors also evaluated the severity of OCD of patients [Clinical Global Impression-Severity (CGI-S)] (31). All secondary outcomes were evaluated at baseline and post-intervention.

#### Instruments

The Y-BOCS consists of 10 items that assess the severity of obsessions and compulsions using a five-point Likert scale (32, 33). The Cronbach’s alpha for the Japanese version of the Y-BOCS is 0.89 (25). The Japanese version of the Self-Report Y-BOCS was also collected at each session with the patient. The Cronbach’s alpha for the Japanese version of the Self-Report Y-BOCS is 0.90 (34).

The MINI-International Neuropsychiatric Interview (MINI) is a structured diagnostic interview for DSM-IV and ICD-10 psychiatric disorders (35). The Japanese version of the MINI is reliable and valid (36).

The EuroQol (EQ-5D-3L) is a five-item self-report questionnaire that measures health-related quality of life (QOL) across five domains: mobility, self-care, usual activities, pain and/or discomfort, and anxiety and/or depression (37). The Japanese version of the EuroQol is reliable and valid (29).

The SDS is a three-item self-report inventory for assessing functional impairment at work or school as well as in social and family life using a 10-point Likert scale (38). The Cronbach’s alpha for the Japanese version of the SDS is 0.87 (28).

The K6 is a five-item self-report screening scale for psychological distress that uses a five-point Likert scale (39). The Cronbach’s alpha for the Japanese version of the K6 is 0.85 (27).

The FAS-SR is a 19-item self-rated questionnaire that measures the frequency of family accommodation in relatives’ responses to OCD symptoms. Relatives are asked to evaluate the frequency of 19 accommodation behaviors (40). The Japanese version of the FAS-SR demonstrated good internal consistency and test–retest reliability (30).

The Family Accommodation Scale for OCD Patient-Rated version (FAS-PV) is a 19-item self-rated questionnaire that measures the frequency of accommodating behaviors carried out by the relative. The FAS-PV scores demonstrated good psychometric properties and validity in a recent study (41).

The CGI-S and PGI-S assess global severity level based upon observed and reported symptoms, behaviors, and functioning in the past 7 days (31).

The BDI-II is a 21-item self-report questionnaire that measures the severity of depressive symptoms in the past 2 weeks (42). The Japanese version of the BDI-II demonstrated excellent internal consistency and item homogeneity (26).

### Statistical Methods

Data were analyzed using R 3.3.2 (R Core Team, 2016, Vienna, Austria). To test for significant group differences, we analyzed outcomes using linear mixed models. The Y-BOCS score was considered the primary dependent variable. Group allocation, assessment period (baseline and post-intervention), and the interaction between group allocation and assessment period were regarded as fixed-effect factors, whereas the participants were regarded as random-effect factors. Data from all allocated participants were analyzed with intention-to-treat (ITT) principles. Secondary outcomes were analyzed in the same way as primary outcomes. For all analyses, statistical significance was set at *P* < 0.05. The between-group standardized mean difference (Hedges’ *g*) at post-treatment from the linear mixed model was adjusted for gender, age, and disorder duration. The within-group standardized mean differences from pre- to post-statement (Hedges’ *g*) from the linear mixed model were adjusted for gender, age, and disorder duration. The sample size of this study was determined in reference to previous reports of pilot randomized trials for OCD study (14, 43).

## Results

**Figure 1:**
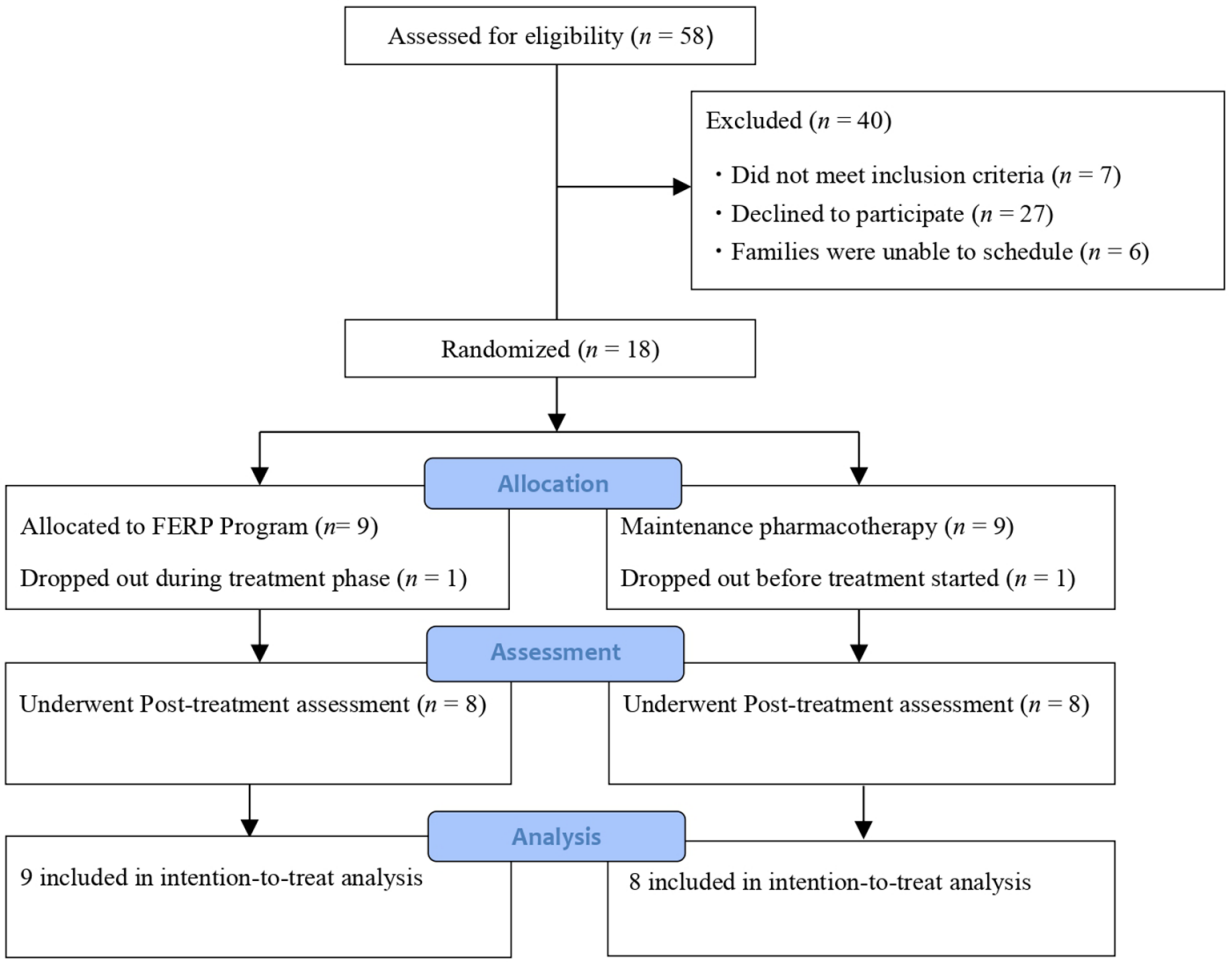
Recruitment flowchart following CONSORT guidelines.

**Figure 1** shows the study process based on the CONSORT guidelines. We screened 58 OCD patients and their family members, of which 18 met the inclusion criteria and were randomized to receive FERP plus TAU (FERP group, *n* = 9) or TAU (control, *n* = 9).

**Table 2** shows the sociodemographic and clinical characteristics of the study participants at baseline. Family members who participated in the study were 13 parents (76.5%), three spouses (17.6%), and one partner (5.9%).

**Table 2:** Participant characteristics at baseline by group.

Characteristics	Full sample (*N* = 17)	FERP (*n* = 9)	Control (*n* = 8)
Patient			
Age, mean (SD), years	30.12 (9.0)	29.44 (8.3)	30.88 (10.1)
Female, *n* (%)	8 (47.1)	3 (33.3)	5 (62.5)
Education, *n* (%)			
University or higher	8 (47.1)	4 (44.4)	4 (50.0)
High school	7 (41.2)	3 (33.3)	4 (50.0)
Others	2 (11.7)	2 (22.2)	0 (0.0)
Unemployed, *n* (%)	14 (82.4)	6 (66.7)	8 (100.0)
Marital status, *n* (%)			
Married	9 (52.9)	2 (22.2)	7 (87.5)
Unmarried	8 (47.1)	7 (77.8)	1 (12.5)
Age at onset, mean (SD), years	20.1 (8.5)	23.2 (8.1)	16.5 (7.9)
Duration, mean (SD), years	10.2 (7.6)	6.4 (6.7)	14.5 (6.3)
Cohabiting, *n* (%)	17 (100.0)	9 (100.0)	8 (100.0)
Previous hospitalization, *n* (%)	16 (94.1)	8 (88.9)	8 (100.0)
Previous suicide attempt, *n* (%)	0 (0.0)	0 (0.0)	0 (0.0)
Family history of psychiatric disorders, *n* (%)	3 (17.6)	1 (11.1)	2 (22.2)
Duration of obsessive-compulsive disorder episode, mean (SD), months	122.2 (91.0)	76.2 (80.5)	174.0 (75.6)
Comorbid DSM-IV Axis I diagnosis, *n* (%)			
Depressive disorder	4 (23.5)	3 (33.3)	1 (12.5)
Other mood disorder	2 (11.8)	1 (11.1)	1 (12.5)
Agoraphobia	3 (17.6)	1 (11.1)	2 (25.0)
Generalized anxiety disorder	3 (17.6)	2 (22.2)	1 (12.5)
Taking psychiatric medication at baseline, *n* (%)	15 (88.2)	8 (88.9)	7 (87.5)
Referred to the study by, *n* (%)			
Treating psychiatrist	0 (0.0)	0 (0.0)	0 (0.0)
Family	0 (0.0)	0 (0.0)	0 (0.0)
Self	17 (100.0)	9 (100.0)	8 (100.0)
Family member			
Age mean (SD), years	53.7 (11.2)	50.9 (12.9)	56.8 (8.8)
Female, *n* (%)	15 (88.2)	7 (77.8)	8 (87.5)

The symptom severity of OCD for participants at baseline was severe (YBOCS total score = 27.2, SD = 4.3) and depressive symptom severity was moderate (BDI-II total score = 25.7, SD = 9.7). The psychological distress score for both patients and their family members were higher than the cutoff score, indicating the existence of high psychological distress [K6 total score (patient) = 20.1, SD = 5.4; K6 total score (family member) = 15.8, SD = 4.7]. The functional impairment was moderate to markedly low (work/school on SDS = 7.9, SD = 2.1; social life on SDS = 7.7, SD = 2.1; family life/home responsibilities on SDS = 6.7, SD = 2.4). The frequency of family accommodation was severely high (FAS-SR total score = 28.8, SD = 12.7; FAS-PV total score = 24.7, SD = 14.5).

**Table 3** summarizes the primary outcome measures by treatment group. For primary outcomes, the between-group mean difference of Y-BOCS in the FERP group minus control group at post-intervention was significant [standardized mean difference (Hedges’ *g*), −1.58; 95% CL, −2.72 to −0.44; *P* < 0.001]. The within-group mean difference of Y-BOCS in the FERP post group minus FERP pre group was significant [standardized mean difference (Hedges’ *g*), −1.56; 95% CL, −2.69 to 0.0.42; *P* < 0.001].

**Table 3:** Summary of primary and secondary outcomes.

		FERP	Control	Standardized between-group difference^a^ (95% CI)		*P* value
		(*n* = 9), *n* (SD)	(*n* = 8), *n* (SD)		
**Primary outcome**						
YBOCS total	Pre	26.67 (5.50)	27.75 (3.24)			
	Post	16.62 (6.19)	26.38 (4.72)	−1.58	[−2.72, −0.44]	0.000
	Standardized within-group effect size [95% CI]^b^	−1.56 [−2.69, −0.42]	−0.23 [−1.24, 0.79]			
YBOCS compulsion	Pre	13.67 (3.57)	13.75 (2.19)			
	Post	9.12 (2.59)	13.38 (2.39)	−1.24	[−2.32, −0.16]	0.002
		−1.29 [−2.38, −0.20]	−0.12 [−1.13, 0.90]			
YBOCS obsession	Pre	13.00 (2.24)	14.00 (1.69)			
	Post	7.50 (3.70)	13.00 (2.83)	−1.7	[−2.86, −0.54]	0.000
	Standardized within-group effect size [95% CI]^b^	−1.61 [−2.76, −0.47]	−0.31 [−1.33, 0.71]			
**Secondary outcome**						
*Global impression severity*						
PGI severity	Pre	5.25 (0.89)	5.25 (1.16)			
	Post	3.62 (0.92)	4.88 (1.55)	−0.74	[−1.75, 0.28]	0.068
	Standardized within-group effect size [95% CI]^b^	−1.36 [−2.47, −0.26]	−0.30 [−1.32, 0.72]			
CGI severity	Pre	5.44 (0.73)	5.25 (0.89)			
	Post	3.50 (0.76)	5.00 (1.20)	−1.26	[−2.35, −0.18]	0.003
	Standardized within-group effect size [95% CI]^b^	−1.82 [−3.01, −0.63]	−0.24 [−1.26, 0.78]			
*Depressive symptom*						
BDI-II	Pre	24.89 (7.10)	26.50 (13.03)			
	Post	15.12 (7.10)	26.12 (15.97)	−0.71	[−1.72, −0.31]	0.003
	Standardized within-group effect size [95% CI]^b^	−0.79 [−1.82, 0.23]	−0.03 [−1.04, 0.99]			
K6 patient score	Pre	19.86 (4.71)	20.38 (6.61)			
	Post	14.62 (3.58)	18.50 (7.95)	−0.54	[−1.54, 0.47]	0.207
	Standardized within-group effect size [95% CI]^b^	−0.76 [−1.78, 0.26]	−0.28 [−1.30, 0.74]			
*Functional impairment*						
SDS	Pre	22.44 (5.77)	22.00 (6.87)			
	Post	12.38 (8.05)	18.88 (8.68)	−0.61	[−1.62, 0.4]	0.095
	Standardized within-group effect size [95% CI]^b^	−1.18 [−2.25, −0.11]	−0.37 [−1.40, 0.65]			
*Quality of life*						
EQ5D	Pre	0.56 (0.28)	0.57 (0.19)			
	Post	0.69 (0.28)	0.57 (0.20)	0.43	[−0.56, 1.43]	0.113
	Standardized within-group effect size [95% CI]^b^	0.57 [−0.44, 1.57]	0.00 [−1.02, 1.01]			
*Family involvement*						
FAS-PV	Pre	20.67 (11.01)	29.12 (18.07)			
	Post	12.25 (10.58)	29.57 (20.60)	−0.81	[−1.83, 0.22]	0.053
	Standardized within-group effect size [95% CI]^b^	−0.50 [−1.50, 0.50]	−0.04 [−1.05, 0.98]			
FAS-SR	Pre	21.89 (10.68)	36.57 (12.47)			
	Post	17.12 (12.48)	39.00 (14.34)	−0.77	[−1.79, 0.25]^c^	0.359
	Standardized within-group effect size [95% CI]^b^	−0.34 [−1.33, 0.66]	0.01 [−1.00, 1.03]			
*Family functioning*						
K6 family score	Pre	14.22 (3.63)	17.56 (5.46)			
	Post	12.00 (3.42)	17.86 (5.64)	−0.55	[−1.56, 0.45]^c^	0.672
	Standardized within-group effect size [95% CI]^b^	−0.29 [−1.28, 0.70]	−0.07 [−1.09, 0.94]			

YBOCS, Yale-Brown Obsessive-Compulsive Scale; PGI, Patient Global Impression; CGI, Clinical Global Impression; BDI-II, Beck Depression Inventory II; K6, Kessler Psychological Distress Scale; SDS, Sheehan Disability Scale; EQ5D, EuroQol; FAS-SR, Family Accommodation Scale for OCD Self-Rated version; FAS-PV, Family Accommodation Scale for OCD Patient-Rated version.

a Between-group standardized mean differences (Hedges’ g) at post-treatment from the linear mixed model adjusted for gender, age, and disorder duration.

b Within-group standardized mean differences from pre- to post-treatment (Hedges’ g) from the linear mixed model adjusted for gender, age, and disorder duration.

c Between-group standardized mean differences (Hedges’ g) at post-treatment from the linear mixed model adjusted for gender, age, disorder duration, and baseline outcome score.

Two (22.2%) of the patients in the FERP group reached remission, while no patients in the control group achieved remission. Five patients (55.6%) in the FERP group achieved treatment response, with no treatment response in the control group. **Table 3** also includes the between-group mean difference of the secondary outcome measures in the FERP group minus control group at post-intervention. There was a large, significant effect for CGI, as well as a moderate but significant effect for PGI, BDI, SDS, and EQ5D. Detailed estimates of all variables from LMM for all outcomes are reported in **Table S1**. The effect for family outcome [FAS-PV, FAS-SR, and K6 (family)] was not significant from LMM.

The dropout rate for this study was 11.8% (2/17). One participant dropped out prior to the commencement of the intervention, and another withdrew due to a change in the patient’s work schedule. None of the participants experienced serious adverse events during the intervention period.

## Discussion

Our findings show that the addition of the FERP program to TAU was effective in reducing OCD symptoms. Additionally, depressive symptoms were significantly alleviated in the FERP group. Furthermore, improvements of QOL, social functioning, and global impression of severity were also observed. These results partially support our hypothesis that FERP would result in greater improvement of OCD symptom severity compared to the control group.

In this study, a large effect size was observed with the Y-BOCS. There are several possible reasons for it. First, the FERP program components may be appropriate for Japanese participants. In particular, focusing on practicing for both patients and families to acquire communication skills and including a family member as a coach for the patient—such as for reducing accommodation and encouraging the patient to not disrupt home-based ERP—may be effective for fostering patients’ motivation and reducing OCD symptoms. The program components were almost the same as in previous US-based studies, which have been reported to result in significant reduction of OCD symptoms (14, 15, 44). However, there are considerable differences between the US and Japan in terms of family communication styles (45). Further research that focus on Japanese family functioning and communication styles to develop improved family interventions for OCD are necessary. Second, the FERP program included an additional component related to the development of an action plan for change using a family contract, which was not included in previous randomized controlled trials. The contracting process was included as it may help improve communication about important issues, family roles, and needs (46). Third, the FERP program included joint sessions with the patient and family member, enabling them to share their emotions with each other and identify their goals and strategies for overcoming OCD as a team. Such a team approach may be effective in the treatment of OCD. “Japanese families often describe caring for their mentally ill family members as a ‘lonely battle’” (47). The stigma of mental illness in Japan may lead to such isolation outside the home (48). Joint sessions, a team approach, and normalization of their emotions, as utilized in this study, may improve their sense of isolation and resistance to sharing their family member’s OCD diagnosis with others. Indeed, we received positive feedback from the family members on these aspects, such as “Communication skill exercise was really helpful,” “All educational sheets were very useful,” and “I understood the difference between reacting and responding to OCD moments.”

The dropout rate of the present study (11.8%) was lower than the rate (19.1%) found in an earlier meta-analysis (49). There are several possible reasons for this. First, due to the shortage of trained therapists specializing in OCD treatment and limited psychiatric consultation time, participants may have had strong expectations from the FERP program. Indeed, all participants were referred not by their treating psychiatrists but the patients themselves in search of optimal care. Due to the Japanese medical insurance system, psychiatric consultation times are limited (50). Although the first-line treatment for OCD includes CBT and pharmacological treatment, OCD patients in Japan do not get many opportunities to receive ERP. Second, patients in this study may have developed a stronger treatment engagement. The rate of homework implementation was very high, and the average of general evaluation for competency, ranging from 0 to 5, was 4.0. In particular, the average score for the quality of rapport, such as warmth, openness, respect, and humor, was 4.2. Third, the sample size in this study was notably lower than larger randomized controlled trials. Attention should be given to the dropout rate in future research.

Contrary to our hypothesis, family functioning, including levels of family accommodation and psychological distress, were not significantly improved in the FERP group compared to the control group in this study. While confidence intervals were wide and contained zero, point estimates of effect size were large to moderate in these family outcomes. As symptom improvement for OCD patients was the primary outcome in this study, random assignment was applied only to patient placement and not family in this study. As a result, there were differences between groups in the family-related factors at the baseline, which may contribute to the increased uncertainty of the family effect estimate. Therefore, future research that sets family function or mood as the primary outcome and random assignment of the family members is necessary for balancing family factors.

In the present study, we intended to examine patients who had at least a mild severity of OCD symptoms at the study entry. The reasons for focusing on such patients are that mild symptoms are 1) associated with impairment of QOL and functioning (2), and 2) linked with the recovery stage of OCD (defined as a score of less than 8 on the Y-BOCS) (51). Although we intended to include patients who had at least a mild severity of symptoms, the mean baseline Y-BOOS score of this sample indicated to be 27.2, which referred to moderate–severe OCD symptoms (52). Thus, the OCD symptom severity of our sample shown was similar to previous researches (14, 15, 53).

This study has several limitations. First, the sample size was small. As this study was conducted at fixed times, the study population was limited and selection bias may have occurred. Although recruitment was limited, statistical analysis in this study was performed with appropriate statistical power. Second, in this study, 69.0% of the study participants were excluded after they were assessed for eligibility. Of the excluded participants, 27 (67.5%) did not want to participate in a randomized trial as they wanted to start CBT for OCD as soon as possible. Furthermore, six (15.0%) of the excluded participants were family members who were unable to schedule the requisite number of sessions. Moreover, the study participants were recruited from only two sites, which may limit the generalizability of the findings. Third, as there was no follow-up assessment, we were unable to evaluate the prognosis for this study. Fourth, this study used waiting list controls rather than active controls. Participants in the control group were required to have the same number of sessions as the intervention group. Fifth, although we used randomization to ensure a good balance of patients and clinical characteristics, including pharmacotherapy, we could not totally control for medication status because we used treatment-as-usual for both the FERP and control groups.

## Conclusion

In conclusion, the present study shows that the FERP program is effective in alleviating OCD symptoms, reducing the frequency of family accommodation and improving family functioning with adult outpatients with OCD and their family members. To our knowledge, this is the first randomized clinical trial that examines the feasibility and efficacy of the family-based ERP program in Japan. Additional trials are needed to replicate our results with a reasonably large sample size, recruiting participants from several sites and spanning a wider geographical area in Japan before a definite conclusion is drawn.

## Data Availability Statement

The datasets generated for this study are available on request to the corresponding author.

## Ethics Statement

The studies involving human participants were reviewed and approved by: 1. The Ethics Committee of the National Center of Neurology and Psychiatry (Approved number: A2015-018), 2. The Ethics Committee of Keio University Hospital (Approved number: 20180062). The patients/participants provided their written informed consent to participate in this study.

## Author Contributions

All authors contributed to designing the intervention program. AK obtained funding and designed the study. YK obtained finding, refined the study protocol, and was responsible for study management, recruitment, and drafting of the manuscript. MH provided CBT expertise and supervised the therapists. YT conducted the statistical analyses. NH and MM were responsible for monitoring adverse events. IS and NN contributed to the study design, administrative tasks, and material development. KY and CN contributed in developing the framework for training. AN contributed to the study design, drafting of the manuscript, and critical review of the manuscript for important intellectual content. All authors have reviewed and approved the final manuscript.

## Funding

This study was supported by JSPS KAKENHI, Grant Number JP15K17320 (Grant-in-Aid for Young Scientists (B)), MEXT KAKENHI Grant number JP17K04482 (Grant-in-Aid for Scientific Research (C)), and Mental Health Okamoto Memorial Foundation. The funders had no role in the design or conduct of the study, data collection, or and interpretation, decision to publish, or writing of the manuscript.

## Conflict of Interest

The authors declare that the research was conducted in the absence of any commercial or financial relationships that could be construed as a potential conflict of interest.
