# Supplementary material for: An Evaluation of Family-Based Treatment for OCD in Japan: A Pilot Randomized Controlled Trial
Source: Front Psychiatry. 2020 Jan 9;10:932. doi: 10.3389/fpsyt.2019.00932 (PMC6962241; doi:10.3389/fpsyt.2019.00932)
Supplement: Supplementary file 1 [file Table_1.pdf]

Supplementary Material

Table S1

Table S1: Estimates of Fixed effect in each predictor by outcomes from the linear mixed model

Outcomes	Predictors	Estimate	[95%CI]	*p*
YOBOCS total	(Intercept)	23.38	[12.38, 34.37]	0
	Gender	-2.02	[-7.17, 3.13]	0.44
	Age	0.21	[-0.12, 0.54]	0.21
	Duration	0	[-0.04, 0.04]	0.87
	Time (ref: baseline)	-9.4	[-11.55, -7.25]	0
	Group (ref: Control group)	1.69	[-4.40, 7.77]	0.59
	time * group	8.02	[4.98, 11.07]	0
YOBOCS Compulsion	(Intercept)	13.44	[7.65, 19.24]	0
	Gender	-1.9	[-4.59, 0.80]	0.17
	Age	0.09	[-0.09, 0.26]	0.33
	Duration	0	[-0.02, 0.02]	0.83
	Time (ref: baseline)	-4.17	[-5.58, -2.76]	0
	Group (ref: Control group)	0.29	[-2.96, 3.53]	0.86
	time * group	3.8	[1.79, 5.80]	0
YOBOCS Obsession	(Intercept)	9.97	[4.07, 15.86]	0
	Gender	-0.12	[-2.87, 2.64]	0.93
	Age	0.12	[-0.05, 0.30]	0.18
	Duration	-0.01	[-0.03, 0.02]	0.62
	Time (ref: baseline)	-5.24	[-6.46, -4.03]	0
	Group (ref: Control group)	1.39	[-1.88, 4.66]	0.41
	time * group	4.24	[2.52, 5.96]	0
PGI severity	(Intercept)	3.12	[1.17, 5.08]	0
	Gender	0.39	[-0.49, 1.27]	0.39
	Age	0.06	[0.00, 0.12]	0.07
	Duration	0	[-0.01, 0.01]	0.81
	Time (ref: baseline)	-1.68	[-2.60, -0.75]	0
	Group (ref: Control group)	-0.39	[-1.68, 0.90]	0.55
	time * group	1.3	[0.02, 2.59]	0.05
CGI severity	(Intercept)	4.86	[3.10, 6.61]	0
	Gender	-0.32	[-1.12, 0.48]	0.44
	Age	0.03	[-0.02, 0.08]	0.27
	Duration	0	[0.00, 0.01]	0.63
	Time (ref: baseline)	-1.88	[-2.52, -1.23]	0
	Group (ref: Control group)	-0.3	[-1.32, 0.73]	0.57

	time * group	1.63	[0.71, 2.54]	0
BDI-II	(Intercept)	12.61	[-11.30, 36.53]	0.3
	Gender	-1.67	[-12.91, 9.57]	0.77
	Age	0.44	[-0.28, 1.15]	0.23
	Duration	0.02	[-0.07, 0.11]	0.63
	Time (ref: baseline)	-10.31	[-14.12, -6.49]	0
	Group (ref: Control group)	-0.59	[-13.72, 12.54]	0.93
	time * group	9.93	[4.53, 15.33]	0
K6 patient score	(Intercept)	15.56	[3.37, 27.75]	0.01
	Gender	-2.51	[-8.15, 3.12]	0.38
	Age	0.23	[-0.13, 0.59]	0.21
	Duration	0.01	[-0.04, 0.05]	0.75
	Time (ref: baseline)	-5	[-8.45, -1.55]	0
	Group (ref: Control group)	0.54	[-6.32, 7.40]	0.88
	time * group	3.13	[-1.48, 7.73]	0.18
SDS	(Intercept)	11.34	[-2.83, 25.51]	0.12
	Gender	1.8	[-4.66, 8.26]	0.58
	Age	0.29	[-0.14, 0.73]	0.19
	Duration	0	[-0.05, 0.05]	0.96
	Time (ref: baseline)	-9.81	[-14.98, -4.65]	0
	Group (ref: Control group)	-1.51	[-9.78, 6.77]	0.72
	time * group	6.69	[-0.65, 14.02]	0.07
EQ5D	(Intercept)	0.21	[-0.31, 0.73]	0.42
	Gender	0.12	[-0.12, 0.36]	0.34
	Age	0.01	[-0.01, 0.02]	0.3
	Duration	0	[0.00, 0.00]	0.45
	Time (ref: baseline)	0.17	[0.03, 0.30]	0.02
	Group (ref: Control group)	0.04	[-0.26, 0.33]	0.8
	time * group	-0.17	[-0.36, 0.03]	0.09
FAS-PV	(Intercept)	3.07	[-30.36, 36.50]	0.86
	Gender	11.14	[-4.53, 26.81]	0.16
	Age	0.14	[-0.86, 1.13]	0.79
	Duration	-0.02	[-0.14, 0.10]	0.79
	Time (ref: baseline)	-9.05	[-14.31, -3.79]	0
	Group (ref: Control group)	6.62	[-11.66, 24.90]	0.48
	time * group	8.35	[0.65, 16.05]	0.03
FAS-SR	(Intercept)	12.47	[-15.29, 40.23]	0.38
	Gender	5.63	[-7.29, 18.55]	0.39
	Age	0.09	[-0.75, 0.93]	0.84
	Duration	-0.01	[-0.11, 0.09]	0.85
	Time (ref: baseline)	-5.39	[-13.32, 2.54]	0.18
	Group (ref: Control group)	16.04	[0.02, 32.06]	0.05
	time * group	5.62	[-5.95, 17.18]	0.34

K6 family score	(Intercept)	7.87	[0.70, 15.04]	0.03
	Gender	-2.85	[-6.10, 0.41]	0.09
	Age	0.37	[0.15, 0.59]	0
	Duration	-0.01	[-0.04, 0.02]	0.43
	Time (ref: baseline)	-1.3	[-4.30, 1.70]	0.39
	Group (ref: Control group)	4.65	[0.36, 8.94]	0.03
	time * group	0.97	[-3.41, 5.35]	0.66
